# The Influence of the Layer Arrangement on the Distortional Post-Buckling Behavior of Open Section Beams

**DOI:** 10.3390/ma13133002

**Published:** 2020-07-06

**Authors:** Tomasz Kubiak, Mariusz Urbaniak, Filip Kazmierczyk

**Affiliations:** Department of Strength of Materials, Faculty of Mechanical Engineering, Lodz University of Technology, 90-924 Lodz, Poland; mariusz.urbaniak@p.lodz.pl (M.U.); filip.kazmierczyk@dokt.p.lodz.pl (F.K.)

**Keywords:** laminate tailoring, thin-walled structures, post-buckling behavior, finite element method, experimental tests

## Abstract

The paper deals with the design of the stacking sequence of layers in the laminate beams with open-cross sections in order to create the desired behavior in the post-buckling range. Laminate beams with channel and lipped channel cross-sections made of glass fiber reinforced polymer (GFRP) laminate with different layer arrangements (symmetrical and nonsymmetrical) have been considered. In case of the nonsymmetrical stacking sequences, hygro-thermally curvature stable (HTCS) laminates have been taken into account. Pure bending was assumed as the type of load. In the case of beams with open cross-sections, this load type can cause the lateral-distortional buckling mode. A parametric study was performed to analyze the influence of layer arrangement on post-buckling behavior. The finite element method was used to developed numerical models and conduct simulations. Additionally, the experimental tests of the channel section beams were performed in order to validate the developed numerical models.

## 1. Introduction

Thin-walled structures made of laminates provide a lot of flexibility in the design of their behavior—called laminate tailoring. The most popular is the layer sequence design to obtain predictable deflection of structures subjected to operating load. Taking into account the typical layer arrangement (i.e., quasi-orthotropic, symmetrical or antisymmetric cross-ply or angle-ply) only in some mentioned above cases the elements of laminate stiffness matrix responsible for coupled behavior (e.g., in-plane with out-of-plane, shear-extension or bending-twisting) leading to the coupled behavior have non-zero value. Laminates with non-standard (i.e., nonsymmetrical) layer arrangements, which can deflect during the manufacturing process (e.g., autoclaving), afford the possibility for different coupled load-deflection behaviors. A solution to this problem is the hygro-thermally curvature stable (HTCS) laminates. A significant amount of investigations dealing with such laminates (generally, plate) exists in the international literature. The following can be mentioned. York analyzed the HTCS design of laminates with non-standard ply angle orientation (+60°, −60°, 0° and 90°) [1]. Furthermore, he analyzed new design methods for tapered laminates under various types of load [2]. Shamsudin and York [3] provide in-depth analyses of standard laminate stacking sequences in order to obtain thermally immune structures. York and Lee [4] performed experimental validation of the proposed numerical model for carbon fiber reinforced polymer (CFRP) laminates with HTCS design. Cui and Li analyzed laminates with bending-twisting coupled structures with hygro-thermal shear distortion (HTSD) immunity [5]. It should be noted that the nonsymmetrical layer arrangement in laminates allows the design of coupling behavior—the literature also deals with this problem, e.g., [6,7].

The influence of the laminate layer arrangement as well as the non-zero elements of laminate coupling stiffness matrix on the behavior of thin-walled structures is presented by many scientists. Debski et al. [8,9] performed compression tests of CFRP C-section columns with different layer orientation. Teter et al. [10] analyzed the effect of the coupling matrix B from A,B,D (A—extensional stiffness submatrix, B—coupling stiffness submatrix D—bending stiffness submatrix) laminate stiffness matrix on the load-carrying capacity of thin walled columns. Rzeczkowski et al. [11] presented an experimental study of the matrix coupling influence on the delamination of CFRP plates. Kolakowski and Mania [12] presented an analysis of the influence of the coupling matrix B for fiber-metal laminate (FML) and columns made of functionally graded material (FGM). They considered the three-mode interactive buckling approach. Cai et al. [13] performed an experimental study of the influence of the type of fiber pattern, i.e., unidirectional, woven on coupling coefficients of glass/epoxy composites. Gao et al. [14] presented the multi-scale method to predict the ABD stiffness matrix in woven composites based on numerical and experimental tests.

The problem considered in this paper focuses on the post-buckling behavior of thin-walled beams subjected to pure bending, where in the case of open cross-section beams the distortional and lateral deflection appears in the post-buckling range. This topic was widely investigated by Camotim et al. [15,16,17] with an analysis of the different modes and their interaction on post buckling behavior. In those papers, different approaches have been employed. Bebiano et al. [15] presented an application of the latest GBTUL 2.0 (Generalised Beam Theory University of Lisbone, Lisbone, Portugal) software. The paper presents the possibilities of the mentioned software in the case of buckling and vibration for thin walled structures. Martins et al. [16] analyzed simply supported steel beams with lipped channel, Z and hat sections under pure bending, using the direct strength method (DSM). In [17], the compression of lipped channel section columns is shown in the form of experimental tests and the DSM design. Similar investigations have been performed by Rasmunsen et al. [18,19], Szymczak and Kujawa [20,21,22] and Magnucki and Paczos [23,24,25].

Considering the above short literature overview, it can be noted that there are no papers dealing with laminate tailoring for the design of buckling and post-buckling behavior. This was the reason why the authors decided to perform a parametric study with different layer arrangements, including non-symmetrical HTCS laminates, in order to check their influence on buckling and post-buckling behavior. Special attention has been paid to the eventual possibility of the reduction of lateral deflection in the post-buckling range. The study has been performed by developing the finite element models, which have been solved using the commercial software ANSYS^®^ version 18.2 [26]. Additionally, the experimental tests were carried out for a few cases in order to validate the proposed numerical model.

## 2. Experimental Tests

### 2.1. Laminate Material Properties Determinations

The material properties of laminate used for beam specimens have been determined in experimental tests [27]. Young moduli in fiber direction E_1_ and in transvers to fiber direction E_2_, Poisson ratio ν_12_ and ultimate tensile stresses in fiber direction X^T^ and in transvers to fiber direction Y^T^ have been determined according to ASTM D 3039 standard [28]. Kirchhoff modulus G_12_ and ultimate shear stress S have been determined in the specimens with layer arrangements ±45° in tensile tests according to ASTM D3518 standard [29]. The ultimate compressive stress in fiber and transvers to fiber direction X^C^, Y^C^ were determined according to ASTM D3410 standard [30]. It should be noted that the most difficult to determine is the ultimate compression stress in fiber direction X^C^, due to the possibility of buckling. However, comparing obtained value with cases presented in literature [31,32,33] it was found that X^C^/X^T^ ratio for unidirectional GFRP material varies from 0.5 to 1.

For each experimental test six specimens have been prepared and tested. The obtained material properties with standard deviations (SD) are presented in Table 1.

### 2.2. Bending Tests of Channel Section Beams

**Figure 1:**
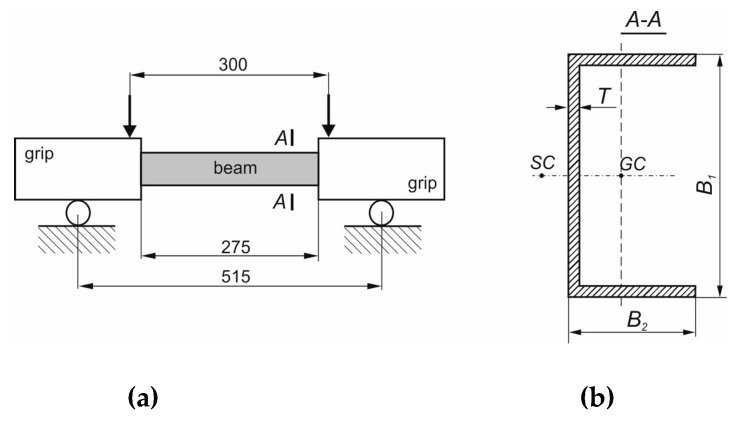
(**a**) Scheme of four-point bending test performed experimentally. (**b**) Considered cross-section.

The experimental four-point bending tests of channel section beams have been performed to validate the developed numerical models, and choose the most interesting cases for conducting the parametric study. The scheme of four-point bending tests with cross-section dimension of considered channel section beam are presented in Figure 1.

The specimens for experimental tests are made of 8 layers of GFRP pre-preg manufactured using the autoclaving technique. The specimens had three following layers arrangements: [0/90/0/90]_S_; [45/−45/90/0]_S_; [45/−45/45/−45]_S_. Nine specimens have been tested—three for each layer layups The cross-section dimensions of all beams have been measured before the test and presented in Table 2 (used notation correspond to those presented in Figure 1).

The experimental tests were conducted on an Instron universal testing machine modernized by Zwick-Roel and equipped with specially designed grips [34]. A scheme of the performed bending test with dimensions describing the span of support and the span of load is shown in Figure 1.

**Figure 2:**
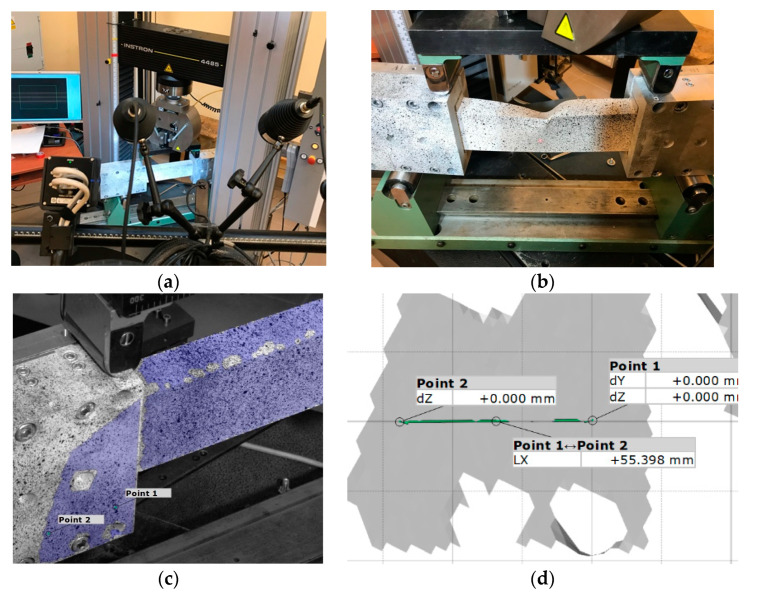
(**a**) Experimental test stand. (**b**) The load and the support in four-point bending test. (**c**) Aramis software view with depicted points (**d**) Zoom of points depicted in left grip.

The tests were performed at a constant velocity of the cross-bar equal to 1.5 mm/min. The values of the loading force applied to the system and the displacement in the points where the load was applied were obtained directly from the machine sensors. In addition, in order to determine deflection of the beam in the entire range of load, a Digital Image Correlation (DIC) technique (Aramis system produced by GOM, Braunschweig, Germany) was used. The data from the DIC were captured with a frequency of 1 Hz together with the load transferred directly from the testing machine. The entire test stand is presented in Figure 2.

The angles of rotation were calculated on the basis of measurements of displacements of two points located on the rigid aluminum grip (see Figure 2c,d). The out of plane displacement (dZ) was used to calculate the horizontal angle of rotation. Vertical displacement of the crosshead of the testing machine was used to calculate the horizontal angle of rotation.

Based on the collected data, the load vs. angle of rotations M_b_ (α_V_) and M_b_ (α_H_) were determined. Additionally, using a 3D optical system, the deflections of the beam were analyzed.

## 3. Numerical Model

The numerical model has been developed by employing the finite element method in the commercial software ANSYS^®^ version 18.2 [26]. The tested beams were subjected to pure bending. The model, i.e., geometry, boundary conditions and the way of load appliance has been assumed as close as possible to those in the experimental test stand (Figure 2). The geometry of the proposed numerical model is very close to those presented in [35].

**Figure 3:**
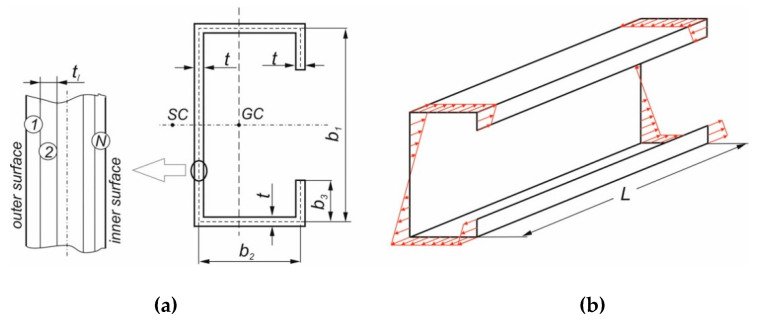
(**a**) The considered laminate beam dimensions with layer arrangement and (**b**) type of the load.

The overall dimensions of the considered beams with the sequence of layer arrangement as well as the type of load are presented in Figure 3. It should be noted that the composite cross-section dimensions in numerical model correspond to the mid-plane of each walls of the laminate beam.

In the performed research, thin-walled channel (b_3_ = 0, see Figure 3) and lip-channel (b_3_ ≠ 0) section beams, made of laminate with different layer arrangements have been considered.

The linear elastic material model has been assumed—all considered materials obey the Hooks’ Law. The material properties of each ply of GFRP laminate are assumed as same as they were determined in experimental tests and presented in Table 1. It should be added that in case when all layers have the fiber direction inclined to the beam’s wall edges ± 45° the nonlinear even plastic behavior [35] appear in far post-buckling range—does not have any influence on buckling load and post-buckling behavior with small deflection. Considering the above, it was decided to not to take it into the numerical model because it may disturb the analysis of influence of the layer arrangements with elements of ABD laminate stiffness matrix on buckling load, as well as post-buckling behavior and post-buckling stiffness.

**Figure 4:**
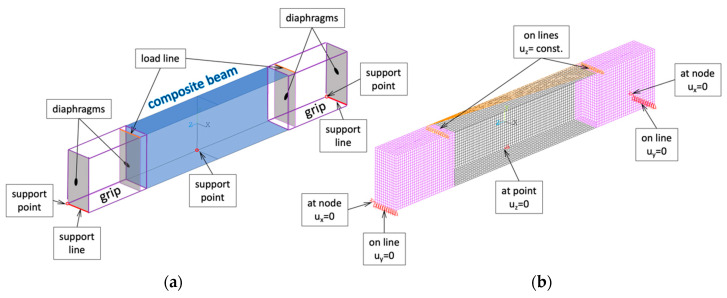
(**a**) Geometry of numerical model; (**b**) discrete model with applied boundary condition and load.

The real grips (Figure 2b) were made of aluminum block [34], but in the numerical model it was decided to model it as a thin-walled beam with a rectangular cross-section (Figure 4) made of steel, with Young modulus E = 200 GPa and Poisson ratio ν_12_ = 0.3.

All the beams were modelled using four-node shell elements with six degrees of freedom at each node (Shell 181, where the first shear deformation theory was introduced). The beams corresponding to the tested specimen are depicted in black in Figure 4b and the parts corresponding to the grips are depicted in purple in Figure 4b. The ways of discretization (the size of elements) are assumed based on earlier experiences with similar numerical calculations [35,36].

The load and the support have been introduced on the grips, which are modeled as a thin-walled structure (see Figure 2b) with rectangular cross-section b_1_ × b_2_ and the same thickness t as the composite beam’s specimen. Having in mind that the real grips are very stiff, additionally in cross-section of numerical model where the load was introduced and the structure was supported the diaphragms have been applied (see Figure 4a). It allowed the avoidance of the occurrence of stress concentrations and unexpected deformations. The geometry of the developed model is presented in Figure 4a, where the lines and points taken for the load and boundary condition introduction are depicted. The assumed boundary condition and the method of load introduction are also presented in the discretized model (see Figure 4b). The boundary conditions have been set as: (i) displacement set to zero at all nodes lying on the lower edges of the end cross-section (u_y_ = 0); (ii) displacement in the horizontal plane in transvers direction to the beam axis at nodes lying in one bottom corner of the end section set to zero (u_x_ = 0); (iii) displacement towards the beam axis set to zero at node located in the mid-span of the beam lying on the edge of bottom flange and the web (see Figure 4).

The load in the developed model was set to the nodes lying on load lines presented in Figure 4a. Additionally, at all the loaded nodes the constant value of vertical displacements (u_z_ = const.) have been set. The vertical force has been used as a load in case of linear buckling analysis and the vertical displacements loaded the system in case of nonlinear analysis. Both types of the load have been set to one node lying along loading lines (Figure 4).

A two-stage solution was considered. Initially the linear buckling analysis was carried out in order to determine buckling modes with corresponding buckling loads. Obtained buckling modes have been used as a shape of initial geometrical imperfection. In the next step, the nonlinear analysis using the Newton–Raphson algorithm was conducted. Only geometrical nonlinearity has been considered—analysis with large displacement. In order to be able to obtain the ultimate load, the analyses were performed with displacement control assuming constant vertical displacement applied on load lines (cf. Figure 4). The force was recalculated from the reactions at node lying on load lines. Knowing the force and the beam with grip dimension the ultimate bending moment has been determined. Initial geometrical imperfections correspond to different buckling modes (the lowest buckling mode or first with the odd number of half-waves) with positive and negative signs of amplitude having been considered. The nonlinear analyses were conducted with and without a progressive damage algorithm with the same parameters as in Gliszczynski and Kubiak [37].

The results of all calculations are presented as a shape of buckling mode and graphs representing the relation between load and deflection. Load is in the form of the bending moment and deflection as the following angles of rotations: α_V_—vertical (in plane of load) beam rotation at support, α_H_—horizontal (lateral to the load plane) beam rotation.

## 4. Numerical Model Validation by the Results of the Experimental Tests

The numerical model validation has been done based on results of experimental tests of channel section beams subjected to pure bending with the following cross-section dimensions (cf. Figure 3): b_1_ = 81 mm, b_2_ = 40 mm, b_3_ = 0 and two different thicknesses t = 1.16 mm for beams with layer arrangement [45/−45/90/0]_S_ and [45/−45/45/−45]_S_ and *t* = 1.2 mm for beams with [0/90/0/90]_S_ lay-ups.

**Figure 5:**
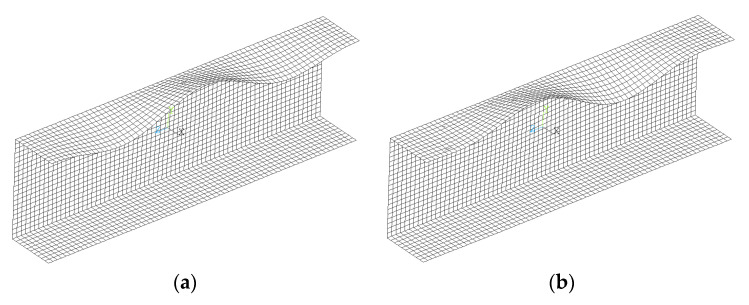
Buckling modes corresponding to the lowest buckling loads for the beams with the layer arrangement: (**a**) [0/90/0/90]_S_ and [45/−45/45/−45]_S_; (**b**) [45/−45/90/0]_S_.

The results of linear buckling analysis (LBA) presenting the buckling modes are shown in Figure 5. In the case of the layer arrangements [0/90/0/90]_S_ and [45/−45/45/−45]_S_, the buckling modes correspond to the lower buckling load are the same (see Figure 5a)—three half-waves of sine on the upper flange. For the beam with stacking sequence [45/−45/90/0]_S_, the first buckling mode has only two half-waves in the longitudinal direction on the upper flange of the beam (cf. Figure 5b). It should be noted that the second buckling mode for this case of layup is characterized by three half-waves, as in the rest of the layer arrangements. Differences between the value of the first and second buckling loads are less than 3% (the second buckling load is 2.8% higher than the first one).

**Figure 6:**
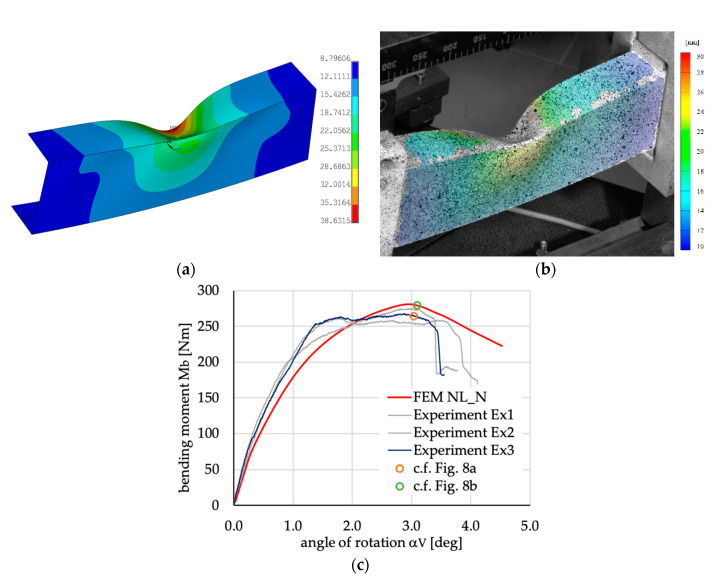
Comparison of beams deflection for case [45/−45/45/−45]_S_ obtained (**a**) numerically with model NL_N; and (**b**) experimentally; (**c**) the load-displacements comparison of curves obtained experimentally and numerically.

The deflection of all beams for the maximal load is very similar. Exemplary results for a beam with layer arrangement [45/−45/45/−45]_S_ obtained numerically and experimentally are presented in Figure 6.

**Figure 7:**
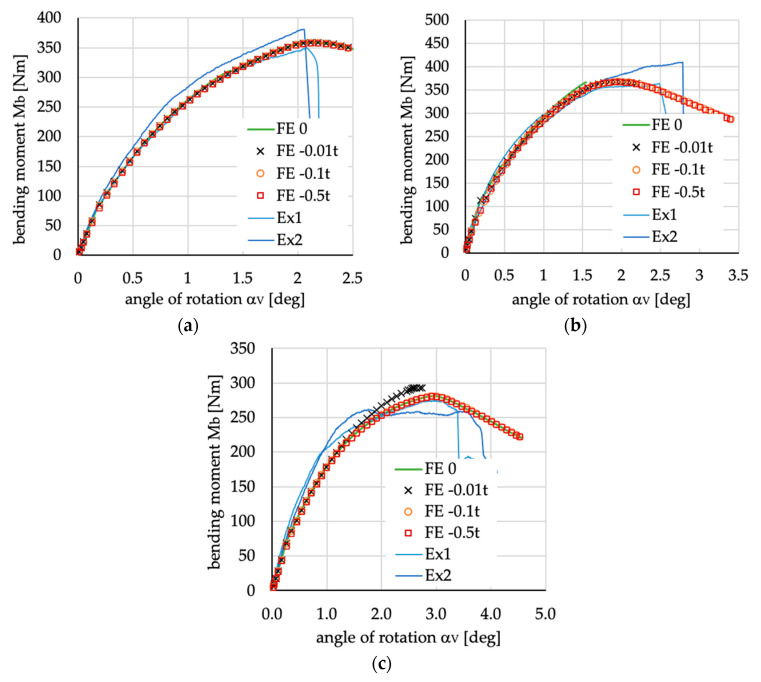
The influence of amplitude of initial geometric imperfection on bending moment vs. angle of rotation α_V_, curves for beams with layer arrangement: (**a**) [45/−45/90/0]_S_; (**b**) [0/90/0/90]_S_; (**c**) [45/−45/45/−45]_S_. FE 0—numerical analysis without imperfection, FE −0.01 t—numerical analysis with the imperfection equal to −1% of thickness, FE −0.1 t—numerical analysis with the imperfection equal to −10% of thickness, FE −0.5 t—numerical analysis with the imperfection equal to −50% of thickness, Ex1—experimental results of first specimen, Ex2—experimental results of second specimen.

Analyzing the beams’ deflection observed during experimental tests, especially when the beam is in the post-buckling range (cf. Figure 6b), it was found that shape of deflection is similar to the buckling mode with three halfwaves of sine in the upper flange (see Figure 5a) assuming negative amplitude of deflection. Taking above into account it was decided to take the buckling mode with three halfwaves (Figure 5a) as a shape of the initial geometric imperfection. The amplitude of initial imperfection was considered as 10% of the beam’s wall thickness [36]. The above assumption is also based on presented in Figure 7 comparison of numerical calculations of beams with different initial imperfection amplitude (curves denoted as: “FE 0”—no imperfection; “FE −0.01 t”—imperfection amplitude equal to 1% of the beam’s wall thickness *t* and negative sign i.e., with direction of deflection opposite to those presented in Figure 5a; “FE −0.1 t”—imperfection amplitude equal to −10% of t; “FE −0.5 t”—imperfection amplitude equal to −50% of t) with results of experimental tests (curves denoted as “Ex1” and “Ex2”). The obtained and presented in Figure 7 results show no significant influence of initial imperfections of amplitude on the course of load-deflection curves. The charts of bending moment vs. angle of rotation for amplitude of initial imperfection equal to 1% (not for case [0/90/0/90]_S_) and 10%, or even 50%, are identical. Considering the beam with layer arrangement [45/−45/90/0]_S_, it can be argued that the models of the beams are too stiff if no initial imperfection or too small amplitudes of geometric imperfection are assumed (cf. Figure 7a).

**Figure 8:**
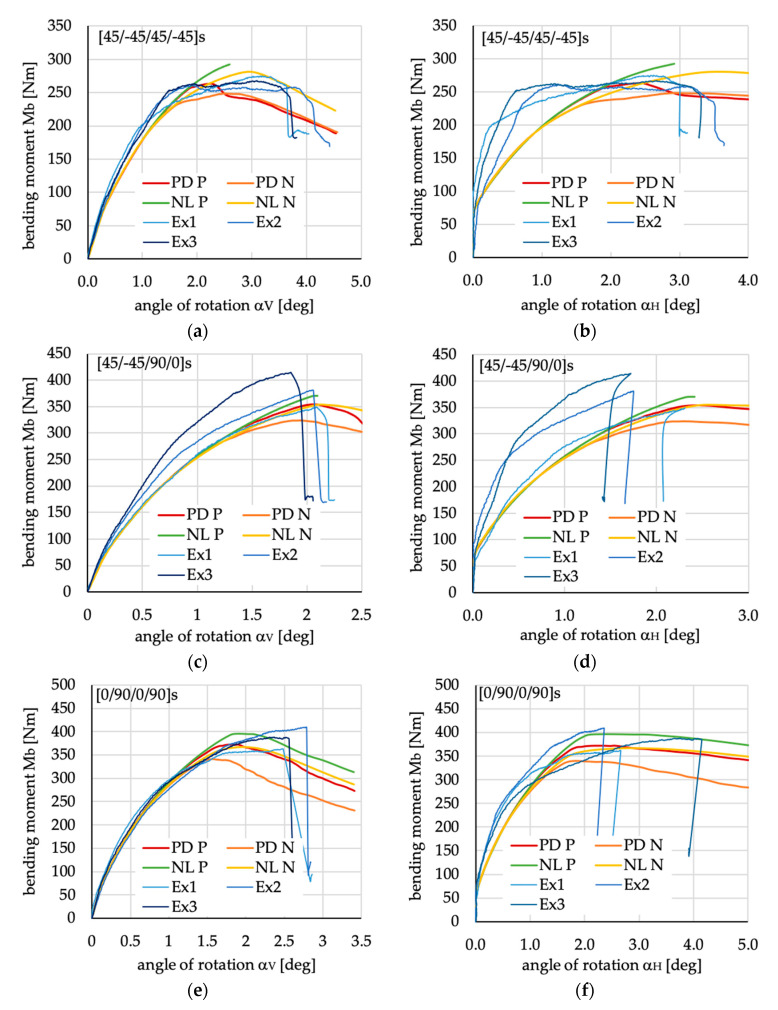
Bending moment vs. angles of rotations M_b_(α_V_) and M_b_(α_H_) for all analyzed cases—comparison of numerical results with experimental ones. (**a**) M_b_(α_V_) for [45/−45/90/0]_S_; (**b**) M_b_(α_H_) for [45/−45/90/0]_S_; (**c**) M_b_(α_V_) for [0/90/0/90]_S_; (**d**) M_b_(α_H_) for [0/90/0/90]_S_; (**e**) M_b_(α_V_) for [45/−45/45/−45]_S_; (**f**) M_b_(α_H_) for [45/−45/45/−45]_S_. PD P—progressive damage algorithm analysis with positive imperfection; PD N—progressive damage algorithm analysis with positive imperfection; NL P—nonlinear analysis with positive imperfection; NL N - nonlinear analysis with negative imperfection; Ex1—experimental results of first specimen; Ex2—experimental results of second specimen; Ex3—experimental results of first specimen.

The comparison of the numerical results with the experimental tests is presented in Figure 8. The type of nonlinear analysis (NL—geometrically nonlinear analysis with linear material properties, PD geometrically nonlinear analysis with progressive damage algorithm PD) and initial geometric imperfections (the sign of amplitude was considered: positive P—direction the same as in Figure 5a, or negative N—direction opposite to the one presented in Figure 5a) have been analyzed and compared with the results of experimental tests.

Analyzing the course of the load-deflection curves presented in the graph in Figure 8, it can be argued that numerical and experimental agreement depends on the type of layer arrangement. It is clear that the numerically obtained bending moment vs. the angle of rotation α_V_ (Figure 8a,c,e) for all analyzed cases in the pre-buckling state, as well as in close post-buckling range are consistent with the experimental results. This is also true when the course of bending moment vs. angle of rotation α_H_ is analyzed for the beam with two types of layer arrangement [45/−45/90/0]_S_ (Figure 8d) and [0/90/0/90]_S_ (Figure 8f). Only in the case of [45/−45/45/−45]_S_ the numerically obtained post-buckling stiffness in the lateral direction to the plane of the load (Figure 8b) differ from the results obtained experimentally.

Comparing the different methods of the numerical calculations (including or excluding the progressive damage algorithm) as well as the sign of amplitude of initial geometric imperfection, it can be noted that when the amplitude of initial imperfection is the same (positive P) as that presented in Figure 5a, the beam behavior in the post-buckling range is slightly too stiff. In some cases (cf. green line in Figure 8a,c), due to convergence difficulties, the solution stops before achieving the assumed load. Assuming the negative sign of initial imperfection amplitude (cases denoted in Figure 8 as PD_N and NL_N), it can be noted that the model ND_N gives the closest results to the experimental tests in comparison to models using the progressive damage algorithm. It should be mentioned that for the beam with layup denoted as [0/90/0/90]_S_, the angle of rotation α_V_ corresponds to the highest bending moment obtained from the numerical calculations and is smaller than that obtained experimentally. The maximal experimental loads and numerical calculations, however, are very close to one another.

Summing up all of the above, it should be mentioned that the results of the experimental tests for specimens with the same layers lay-ups are characterized by diverse course of curves in post-buckling range (especially for case [45/−45/90/0]_S_). Nevertheless, the proposed numerical models predict the beam behavior in the full range of the load (except case [45/−45/45/−45]_S_ layer arrangement—Figure 8a,b), and the obtained results are very similar to those obtained in experimental tests after using the nonlinear numerical model without the progressive damage algorithm, including the worst initial geometric imperfection (NL_N). Additionally, it can be noted that the model NL_N is enough to be employed in the parametric study of layer arrangement influence on buckling load and the post-buckling beam behavior. The above-mentioned model (without the progressive damage algorithm) can exclude the influence of damage in the parametric study. However, It should be noted that in case of [45/−45/45/−45]_S_ layups in far post-buckling range the increase of deflection (both angles of rotations) with constant load value is observed, what could mean the damages or material nonlinearities typical for such a layer arrangement. For this case the numerical model with the progressive damage algorithm (line PD_N in Figure 8a) was better (closer to experimental results) at describing the real load–deflection relation, but the ultimate load, defined as maximal load obtained during the four-point bending test, can be well estimated even by employing the model without the progressive damage algorithm (cf. NL_N curve in Figure 8a).

## 5. Parametric Study

The parametric study was performed for beams with two cross-sections: C-section (b_1_ = 81 mm, b_2_ = 40 mm and t = 1.16 mm) and lipped channel section (b_1_ = 81 mm, b_2_ = 40 mm, b_3_ = 6 mm and t = 1.16 mm) with length L = 275 mm. In the case of the lipped channel section, the width of the stiffeners was assumed in such a way that the lowest buckling mode is distortional [38,39]. The angle of the fiber direction at each layer has been considered as a variable parameter.

### 5.1. Layer Arrangements with Their ABD Laminate Stiffness Matrix

From the mechanics of laminates [31], it is known that it is possible to design the layer arrangement in order to achieve the expected behavior of the components subjected to operating loads. In both considered sections, it is obvious that parts of thin-walled beam (i.e., web, flanges and stiffeners) are subjected to different types of load, which also changes as the load increases. In the pre-buckling range, the web is subjected to in-plane bending, while the flanges are subjected to tension and compression. In the post-buckling range, each part is under combined load, namely subjected to tension and compression as previously and, additionally, to out of plane bending. In order to perform influence analysis of layer arrangement on post-buckling behavior, the possibility of “tailoring” in such a structure and load case has been checked. Considering the classical laminate theory, the relation between internal forces and strains (1) allows to predict the plate behavior under chosen loads.

(1) $$\left\{\begin{array}{c} \left\{\begin{array}{c} N_{x}\\ N_{y}\\ N_{xy} \end{array}\right\}\\ \left\{\begin{array}{c} M_{x}\\ M_{y}\\ M_{xy} \end{array}\right\} \end{array}\right\}=\left[\begin{array}{c} \left[\begin{array}{ccc} A_{11} & A_{12} & A_{16}\\ & A_{22} & A_{26}\\ \mathrm{sym}. & & A_{66} \end{array}\right]\\ \left[\begin{array}{ccc} B_{11} & B_{12} & B_{16}\\ & B_{22} & B_{26}\\ sym. & & B_{66} \end{array}\right] \end{array}\begin{array}{c} \left[\begin{array}{ccc} B_{11} & B_{12} & B_{16}\\ & B_{22} & B_{26}\\ sym. & & B_{66} \end{array}\right]\\ \left[\begin{array}{ccc} D_{11} & D_{12} & D_{16}\\ & D_{22} & D_{26}\\ \mathrm{sym}. & & D_{66} \end{array}\right] \end{array}\right]\left\{\begin{array}{c} \left\{\begin{array}{c} \varepsilon_{x}\\ \varepsilon_{y}\\ \gamma_{xy} \end{array}\right\}\\ \left\{\begin{array}{c} \kappa_{x}\\ \kappa_{y}\\ \kappa_{xy} \end{array}\right\} \end{array}\right\}$$

where [31]: $\left(A_{\mathrm{ij}},B_{\mathrm{ij}},D_{\mathrm{ij}}\right)=\int_{-t/2}^{t/2}{\bar{Q}}_{\mathrm{ij}}\left(1,z,z^{2}\right)\mathrm{dz}$; ${\bar{Q}}_{\mathrm{ij}}$ depends on material properties of ply and its orthotropy axes orientation (fibre orientation θ); z—position of ply respect to the midplane of the plate (beam’s wall); *N*_x_, *N*_y_, *N*_xy_, *M*_x_, *M*_y_, *M*_xy_—internal forces and moments with indexes correspond to midplane with assumed *xy* coordinate system; *ε*_x_, *ε*_y_, *γ*_xy_—strains of the plate (beam’s wall) reduces to the mid-surface; *κ*_x_, *κ*_y_, *κ*_xy_—middle-surface curvatures.

Couplings between in-plane load and out-of-plane responses, or vice-versa, exist when B_ij_ ≠ 0. When A_16_, A_26_ ≠ 0, in-plane shear with extension coupling exists, and in the case when D_16_, D_26_ ≠ 0 out-of-plane bending and twisting coupling appears. According to the international literature (e.g., [1,2,3,4,40]), the used notations for the ABD laminate stiffness matrix are presented in Table 3. In the parametric study, the layer arrangements were assumed in such a way as to check all possible cases of coupling matrix B presented in Table 3.

The cases of layer arrangements considered in the study are presented in Table 4. Some of the chosen layups belong to the hygro-thermal curvature-stable laminates (HTCS) group—their curvature is stable in the manufacturing process [1]. Eight and 16-layer laminates have been considered—in all cases, laminate thickness was the same and equal to 1.16 mm.

The following groups of layups have been considered:

- Laminates with symmetrical layer arrangements—the same three cases as those tested experimentally;
- Arbitrary assumed non-symmetric layer arrangements with given angles of fiber inclinations at each layer, denoted as N1 and N2;
- Non-symmetrical layer arrangements denoted as θ (angle of layer orientation with straight fibers in each layer)—the range of θ from −90 to 90 degrees with a 5-degree step was considered.

**Figure 9:**
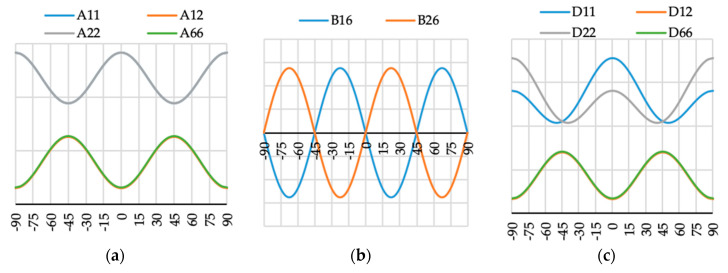
Elements (**a**) [A], (**b**) [B] and (**c**) [D] of laminate stiffness matrix value for case A1(θ).

**Figure 10:**
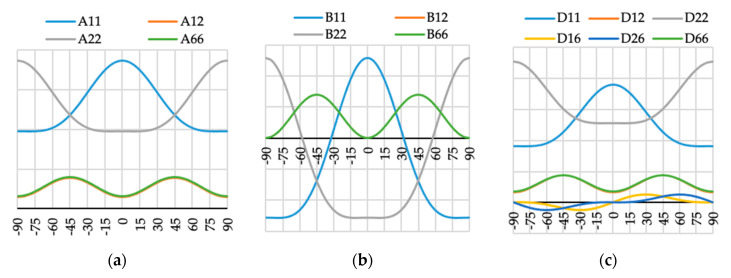
Elements (**a**) [A], (**b**) [B] and (**c**) [D] of laminate stiffness matrix value for case N3(θ).

**Figure 11:**
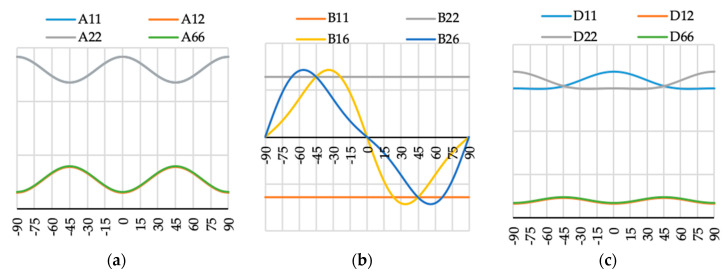
Elements (**a**) [A], (**b**) [B] and (**c**) [D] of laminate stiffness matrix value for case A2(θ).

**Figure 12:**
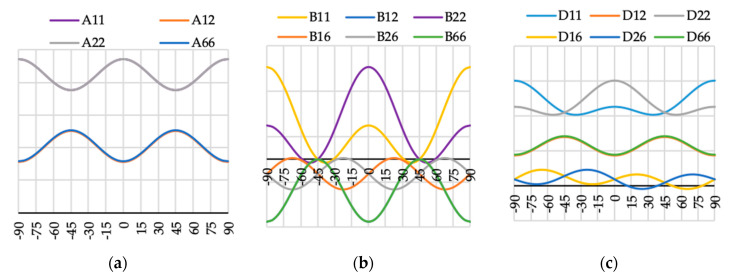
Elements (**a**) [A], (**b**) [B] and (**c**) [D] of laminate stiffness matrix value for case N4(θ).

**Figure 13:**
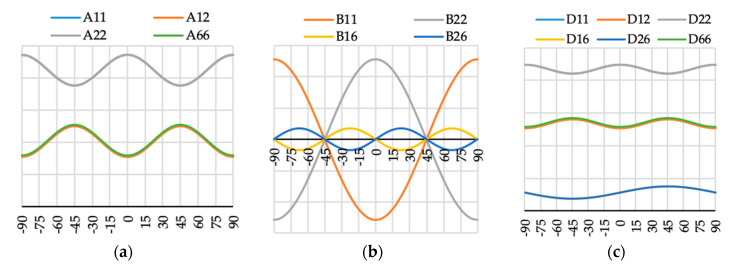
Elements (**a**) [A], (**b**) [B] and (**c**) [D] of laminate stiffness matrix value for case N5(θ).

**Figure 14:**
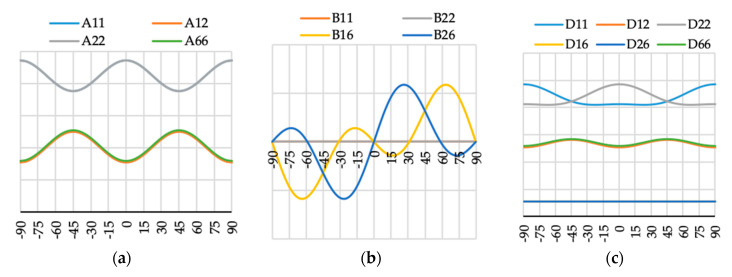
Elements (**a**) [A], (**b**) [B] and (**c**) [D] of laminate stiffness matrix value for case N6(θ).

**Figure 15:**
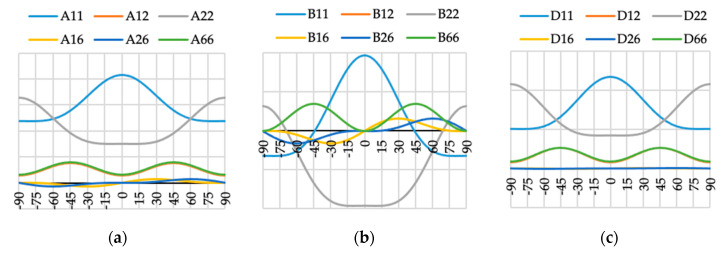
Elements (**a**) [A], (**b**) [B] and (**c**) [D] of laminate stiffness matrix value for case N16(θ).

The variation of ABD laminate stiffness matrix elements for laminates with angle of fiber orientation presented as a variable θ are presented in Figure 9, Figure 10, Figure 11, Figure 12, Figure 13, Figure 14 and Figure 15. These curves have been used in order to choose the angles θ of the layer arrangements with the extremal value of elements in the coupling stiffness matrix, i.e., which could have the highest influence on coupled deflection.

Analyzing how the values of ABD stiffness matrix elements change with the θ angle, it can be noted that for all considered cases all elements except those with indexes “16” and “26” are symmetric with respect to vertical axes (θ = 0). Thus, for laminates which have stiffness matrix denoted as A_S_B_S_D_S_, A_S_B_0_D_S_ and A_S_B***_l_***D_S_ (see Table 4), the change of fiber orientation from positive to negative or vice-versa does not affect laminate behavior. The elements of ABD laminate stiffness matrices with indexes “16” and “26” are antisymmetric with respect to vertical axes (θ = 0) except for D_16_ and D_26_ for layup N4 (cf. Figure 12c).

### 5.2. Layers Arrangement Influence on Load-Deflection Curves 

The relations between bending moment and angles of rotation in two planes: vertical (α_V_)_u_ and horizontal (α_H_)_u_ are presented in Figure 16, Figure 17, Figure 18, Figure 19, Figure 20 and Figure 21 for channel section beams, and in Figure 22, Figure 23, Figure 24, Figure 25, Figure 26 and Figure 27 for lipped section beams.

**Figure 16:**
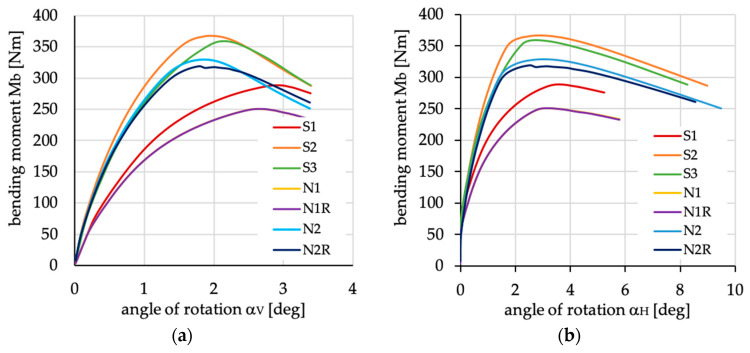
Bending moment vs. angles of rotations in (**a**) vertical and (**b**) horizontal plane for channel section beams with layups denoted as S1, S2, S3, N1, N1R, N2 and N2R.

For both analyzed cases of cross-sections, the relation between the curves presented in Figure 16 and Figure 22 are identical, i.e.,:

- The highest stiffness in the pre-buckling range has been observed for beams with layups denoted as S2, S3, N2 and N2R;
- The highest stiffness in the post-buckling range has been observed for the beam with layup denoted as S2;
- The lowest stiffness in the pre-buckling and post-buckling ranges has been observed for beams with layups denoted as N1 and N1R (the courses of curves are identical);
- The highest ultimate bending moment has been detected for beams with layer arrangement denoted as S2.

The highest stiffness and highest ultimate load with the lowest angle of rotation in both planes was obtained for S2. This case is also presented in the rest of the graphs as the reference curve, for ease comparison.

The beams with all considered antisymmetric layer arrangements and nonsymmetric N5(θ) and N6(θ) for both types of cross-sections have lower stiffness, as well as ultimate load, in the pre-buckling and post-buckling range, than the beam with symmetric layup S2 (cf. Figure 19, Figure 20, Figure 25 and Figure 26). Comparing cases A1(θ), A2(θ), A2R(θ), N5(θ) and N6(θ) with S2, it could be noted that:

- The stiffness of the beams made of laminate with nonsymmetric layups is lower than that of antisymmetric ones;
- The deflection (angle of rotation) in both planes for beams made of antisymmetric and nonsymmetric laminates is higher than for symmetric ones, which is highly visible for lipped section beams (Figure 25 and Figure 26);
- All beams with antisymmetric layups have similar pre-buckling and post-buckling stiffness;
- Beams with both cross-section and nonsymmetric layups N5(θ) and N6(θ) have similar stiffness in the pre-buckling and post-buckling range for θ = ±22.5, ±26.5 and ±63.5;
- The lowest stiffness of all compared beams was observed for those made of laminate denoted as N6(±45).

For the remaining cases of laminates under consideration, i.e., N3(θ) and, N3R(θ) used for channel and lipped section beams, two lay-ups N3(±30) gives the same or very similar results as the beam with layer arrangement S2, while two denoted as N3R(±30) have higher stiffness and higher ultimate load. All of the above proves that the order of the layers in the case of nonsymmetric layups can improve beam behavior.

Analyzing the rest of the cases of lay-ups for channel section beams, it can be noted that all considered layer arrangements denoted as N4(θ) and N4R(θ) give the worst results (i.e., lower ultimate load and lower stiffness in the pre-buckling and post-buckling range) than the beam with lay-up S2. A similar relation can be found when analyzing the results for lipped channel sections (Figure 24) except two cases of laminates denoted as N4(0) and N4(90). For these two-layer arrangements, the lipped channel section beam (cf. burgundy and yellow line in Figure 24) has higher stiffness in the post-buckling range than the beam with layup S2. This can be explained by different beam behavior—the deflection of the compressed flange differs from other cases (cf. Figure 28) because the sense of upper flange deflection is opposite to beam deflection due to bending load (Figure 28a). Additionally, it should be mentioned that, for these cases, the numerical solutions were not completed, and the ultimate loads were not determined.

Taking into consideration ultimate load and beam stiffness in the pre- and post-buckling ranges, the best results were obtained when 16-layered laminates N16(θ) and N16R(θ) were adopted. It should be noted that it was assumed that 8- and 16-layer laminates have the same thickness. The highest stiffness and highest ultimate bending moment were obtained for layups N16R(30) and N16R(-30) in C-section beams and for layer arrangements N16(30) and N16(-30) in lipped section beams. The above-mentioned suggests that the orientation of nonsymmetric laminates is also important, and should be adjusted to the structure (in this case, the beam’s cross-section) alongside the type of load.

In all considered cases, angle α_H_ is increasing significantly when load is around the buckling load, which was also noted in [39].

The deflection of all considered beams with channel cross-sections corresponding to the ultimate load looks exactly the same as those presented in Figure 6. To the contrary, the deflection of the lipped section beam subjected to ultimate or highest load applied is not identical for all cases of laminate layer arrangement and is presented in Figure 28. The deflection presented in Figure 28a corresponds to the cases N4(0) and N4(90) (for which the upper flange is deflected upward and lower flange remains flat), while in Figure 28b corresponds to cases N4(22.5) and N4(-22.5) (it can be seen that both flanges are deflected inwards to the cross-section), in Figure 28c to remaining layer arrangements (both flanges are deflected downwards).

### 5.3. Layers Arrangement Influence on Buckling Load and Ultimate Load

**Figure 17:**
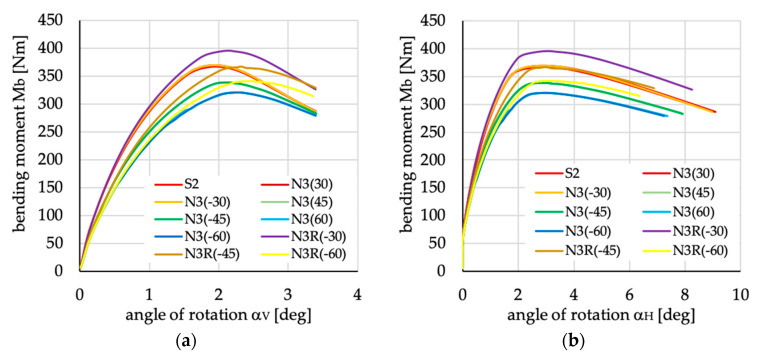
Bending moment vs. angles of rotation in: (**a**) vertical; and (**b**) horizontal planes for channel section beams with layups denoted as N3(θ) and N3R(θ).

**Figure 18:**
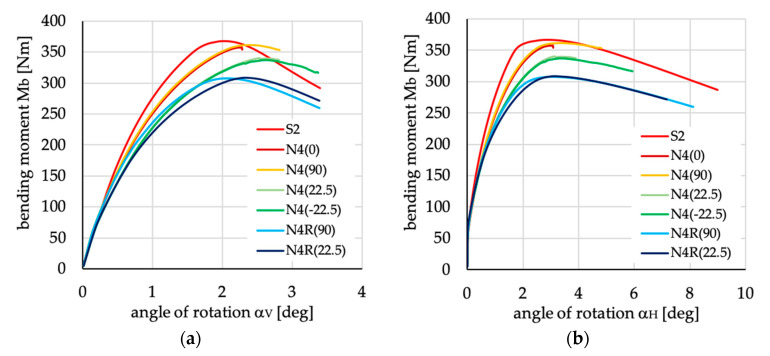
Bending moment vs. angles of rotation in: (**a**) vertical; and (**b**) horizontal planes for channel section beams with layups denoted as N4(θ) and N4R(θ).

**Figure 19:**
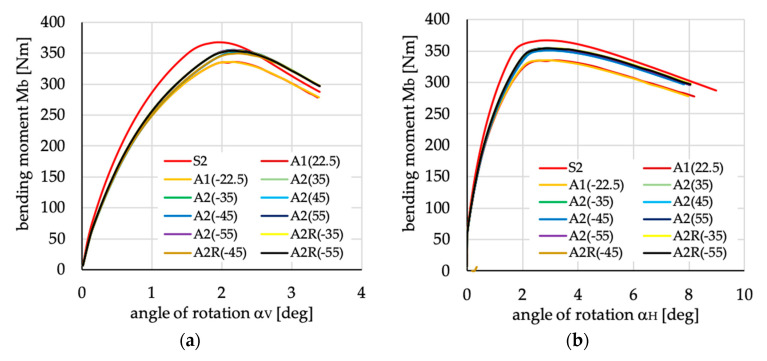
Bending moment vs. angles of rotations in: (**a**) vertical; and (**b**) horizontal planes for channel section beams with layups denoted as A1(θ), A2(θ) and A2R(θ).

**Figure 20:**
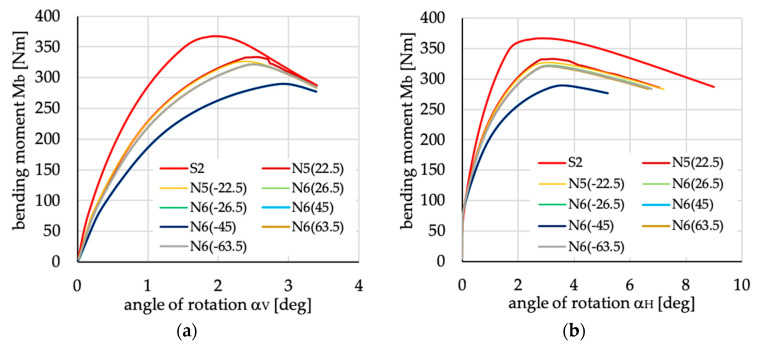
Bending moment vs. angles of rotations in: (**a**) vertical; and (**b**) horizontal planes for channel section beams with layups denoted as N5(θ) and N6(θ).

**Figure 21:**
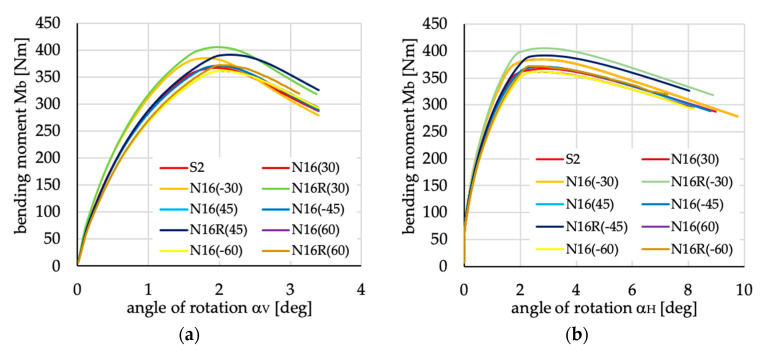
Bending moment vs. angles of rotations in: (**a**) vertical; and (**b**) horizontal planes for channel section beams with layups denoted as N16(θ) and N16R(θ).

**Figure 22:**
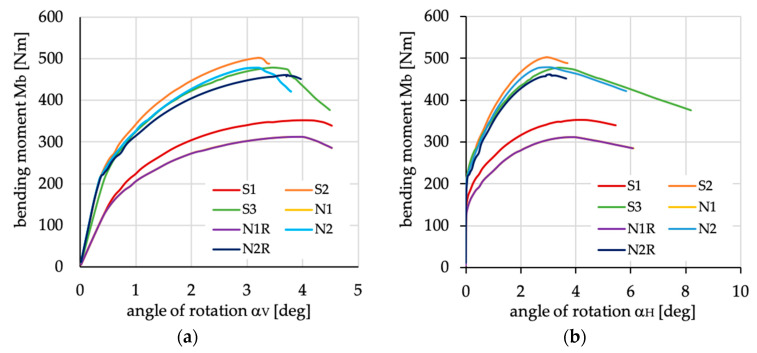
Bending moment vs. angles of rotation in: (**a**) vertical; and (**b**) horizontal planes for lipped section beams with layups denoted as S1, S2, S3, N1, N1R, N2 and N2R.

**Figure 23:**
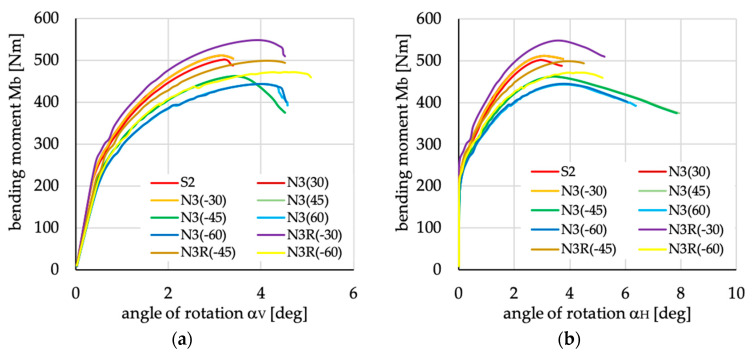
Bending moment vs. angles of rotation in: (**a**) vertical; and (**b**) horizontal planes for lipped section beams with layups denoted as N3(θ) and N3R(θ).

**Figure 24:**
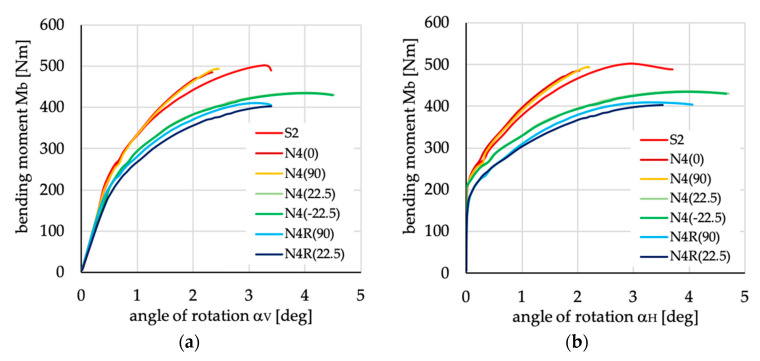
Bending moment vs. angles of rotation in: (**a**) vertical; and (**b**) horizontal planes for lipped section beams with layups denoted as N4(θ) and N4R(θ).

**Figure 25:**
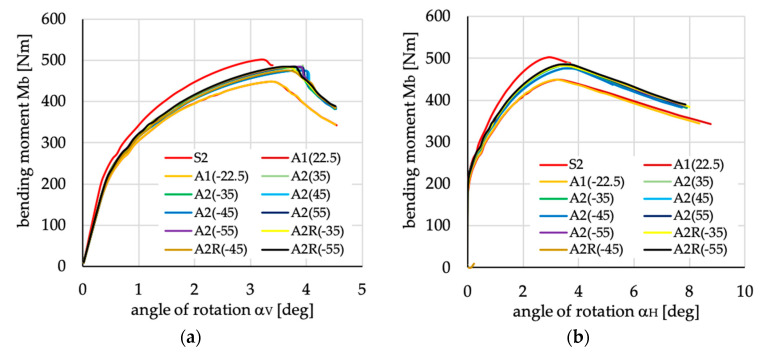
Bending moment vs. angles of rotation in: (**a**) vertical; and (**b**) horizontal planes for lipped section beams with layups denoted as A1(θ), A2(θ) and A2R(θ).

**Figure 26:**
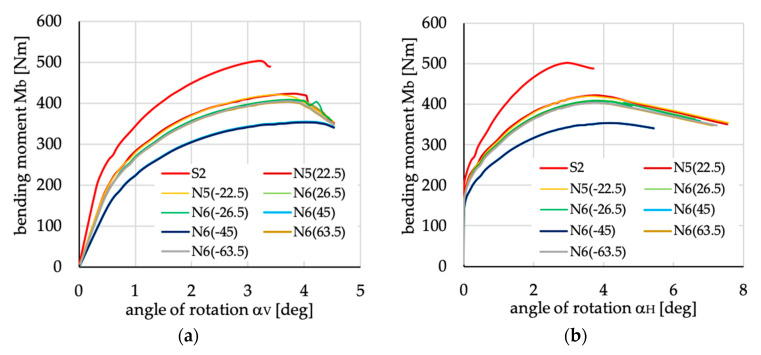
Bending moment vs. angles of rotation in: (**a**) vertical; and (**b**) horizontal planes for lipped section beams with layups denoted as N5(θ) and N6(θ).

**Figure 27:**
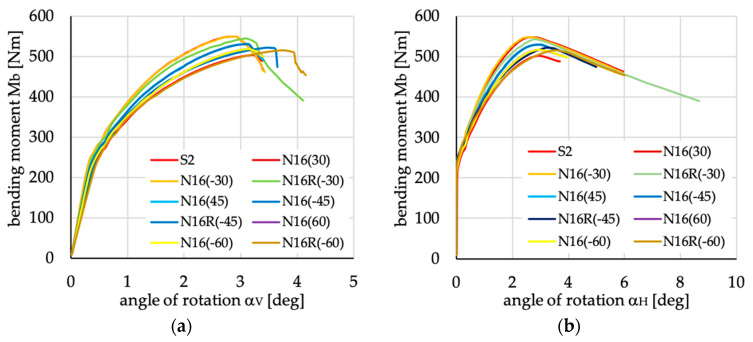
Bending moment vs. angles of rotation in: (**a**) vertical and (**b**) horizontal planes for lipped section beams with layups denoted as N16(θ) and N16R(θ).

The buckling loads M_cr_ determined in the linear buckling analysis and ultimate loads M_u_ from the nonlinear analysis (cf. Figure 16, Figure 17, Figure 18, Figure 19, Figure 20, Figure 21, Figure 22, Figure 23, Figure 24, Figure 25, Figure 26 and Figure 27) with corresponding angles of rotation in two planes, vertical (α_V_)_u_ and horizontal (α_H_)_u_, are presented in Table 5. The extremal values are bolded. It is possible to see that the sign of angle θ does not influence the value of the critical load for both cross-sections. Only in the cases of N4(±22.5) and N5(±22.5) there is a slight difference (cf. Figure 29).

In the case of channel section beams, the highest buckling load was obtained for the beam denoted as S1; moreover, values of (α_V_)_u_ and (α_H_)_u_ are the highest, which leads to relatively low ultimate load. The beam with the layer arrangement denoted as N2R generates the lowest critical load with the corresponding lowest (α_V_)_u_. The highest ultimate load is obtained for the N16R(±30) layer arrangement beam, while the lowest one for the N1 and N1R beams. Similar behavior can be noticed in the case of lipped beams, the ultimate load is also the lowest for the N1 and N1R layups. Furthermore, the mentioned beams indicate the lowest critical load. The highest deformations occur in case N3R(±60), which provides the greatest values of (α_V_)_u_ and (α_H_)_u_. Both angles are the lowest for beam N16(±30), which has the highest load-carrying capacity. Additionally, it was noted that the angles of rotation in both planes corresponding to load-carrying capacity M_u_, in the case of channel section beams for all cases of layer arrangements are in the same relation, i.e., (α_H_)_u_ > (α_V_)_u_. For lipped channel section beams, the relation between angles of rotation (α_H_)_u_ > (α_V_)_u_ depends on layer arrangements and is fulfilled only for cases S1, N4R, N5 and N6. 

## 6. Discussion of Obtained Results

Based on the results presented in Section 5 several relations can be observed.

In both cases of considered cross-sections, the distortional buckling mode was observed. This type of buckling mode leads to lateral deflection in the post-buckling range. In order to find the best layups, i.e., the layup which reduces lateral deflection, the relation between angle of rotation corresponding to ultimate load (α_H_)_u_/(α_V_)_u_ was analyzed. If the relation (α_H_)_u_/(α_V_)_u_ is greater than 1, it means that deflection in lateral direction is higher than in the plane of load. It is obvious that the channel section beams are weaker than lipped section beams; thus, it is only in some layer arrangements of laminate used in lipped section beams that the angles of rotation have a relation lower than one. The lowest value of (α_H_)_u_/(α_V_)_u_ equal to 0.822 was obtained in the case of layup denoted as N2R, but both angles are higher than 3° (cf. Table 5). This means that not only this relation but also the angle of rotation should be checked. Thus, it can be said that the stiffest beam has a lipped cross-section with the layer arrangement denoted as N16(±30).

In Table 6 and Table 7, nondimensional values of stiffness matrices elements ABD are shown for both beam cross-sections. The nondimensional values of elements of the laminate stiffness matrix were obtained after the division of each element by the highest positive value from all analyzed cases, i.e., A_ij_/(A_ij_)_max_, B_ij_/(B_ij_)_max_, D_ij_/(D_ij_)_max_. The maximal values presented in Table 6 and Table 7 are bolded and written in red, and the minimal in blue. The analysis of the values of elements of ABD matrices shows that:

- The extremal buckling load depends on the extremal values of the A_12_, A_66_, D_12_ and D_66_ elements. For channel section beams, the highest buckling load was obtained when the mentioned elements had the highest values, and the lowest buckling load when they had the lowest. The opposite was observed for lipped section beams, i.e., the highest buckling load was obtained when the mentioned elements had the lowest values, and the lowest buckling load for the highest value of those elements.
- The extremal values of load-carrying capacity for both types of considered cross-sections depend on the value of A_11_—if this value is the highest, M_u_ is the highest and if A_11_ is the lowest, M_u_ is the lowest. Additionally, it was found that the maximal ultimate load was obtained for cases when the A_22_ and D_22_ had the lowest value and the extremal A_16_ (maximal negative for channel section and maximal positive for lipped section beams). It has also been found that the minimal ultimate load was obtained in the case when A_12_, A_66_, D_12_ and D_66_ had the maximal values.
- No influence was found in the case of coupling stiffness matrix elements B_ij_, as well as elements D_16_ and D_26_ on the maximal values of buckling and ultimate bending moment. For the minimal ultimate load, it was found that elements B_16_ and B_26_ have extremal negative values, while in the case of minimal buckling load no relations were found.

Comparing the above with the considered cases of layer arrangements, it was noted that N16R(±30) from Table 6 and N16(±30) from Table 7, corresponding to the beams with the highest ultimate load, are characterized by non-zero values of all elements in the D_ij_ stiffness matrix and the largest percentage of layers with 0 angle orientation across the thickness of the laminate. In the case of channel section beams the presence of layers with 45 degree angle (especially at the outer layers) leads to an increase of the critical load and a decrease of the ultimate load. Such a phenomenon is caused by higher D_12_ and D_66_ stiffness matrix elements (S1, N1, N5(±45), N6(±45)).

**Figure 28:**
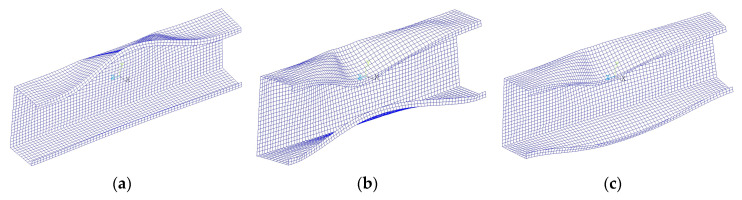
Lipped channel section beam deflection for: (**a**) layups N4(0) and N4(90); (**b**) layups N4(±22.5); (**c**) remaining layer arrangements.

Interesting behavior of the lipped section beam was observed for the layer arrangements denoted as N4(0) and N4(90) (cf. Figure 24a and Figure 28). Due to the fact that in the case of the reverse order of layers—beams N4R(0) and N4R(90)—such a behavior has not been observed, the influence of the coupling stiffness matrix on post-buckling behavior is expected. Additionally, it was noted that for layups N4(90) and N4R(90) the elements of matrices A_ij_ and D_ij_ are identical, while the elements of matrix B_ij_ have opposite signs. As such, it is suggested to check how the deflection changes (vertical displacement of node lying in the mid span of the beam on the edge of the upper flange and stiffener) with load increase for different amplitudes of initial geometric imperfection.

The obtained results for initial imperfection amplitude equaling 10%, 20% and 30% of the wall thickness are presented in Figure 30. The course of equilibrium paths (bending moment vs. vertical displacement of compressed flange—Figure 30a) for different amplitudes of initial imperfection suggest that for small values of imperfection amplitudes (in this case, 10% and 20% of wall thickness) of the wall deflection, due to coupled behavior connected with non-zero values of elements in the **B** laminate stiffness matrix, is “stronger” than deflection connected with the buckling phenomenon. Such a behavior is only observed in this case of layer arrangement, which can be explained by the values of the B_12_ and B_66_ elements, which are the highest of all considered layer arrangements—the nondimensional values of laminate stiffness matrix for case N4(90) are as follows:

(2) $$A=\left[\begin{array}{ccc} 0.70 & 0.62 & 0\\ & 0.81 & 0\\ & & 0.62 \end{array}\right],\;B=\left[\begin{array}{ccc} 0.39 & -1 & -0.12\\ & 0.14 & -0.12\\ & & -1 \end{array}\right],\;D=\left[\begin{array}{ccc} 0.77 & 0.62 & 0.50\\ & 0.62 & 0.41\\ & & 0.62 \end{array}\right]$$

**Figure 29:**
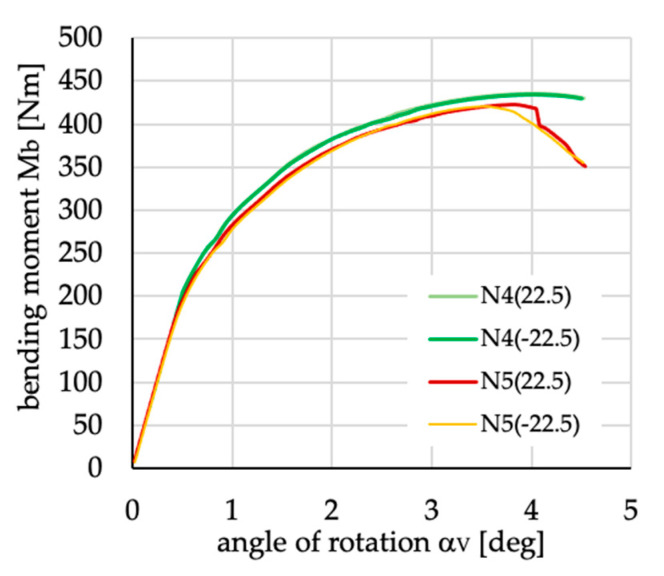
Differences in the course of curves presenting bending moment vs. angle of rotation α_V_ for positive and negative angles of fiber inclination for layups denoted as N4(±22.5) and N5(±22.5).

**Figure 30:**
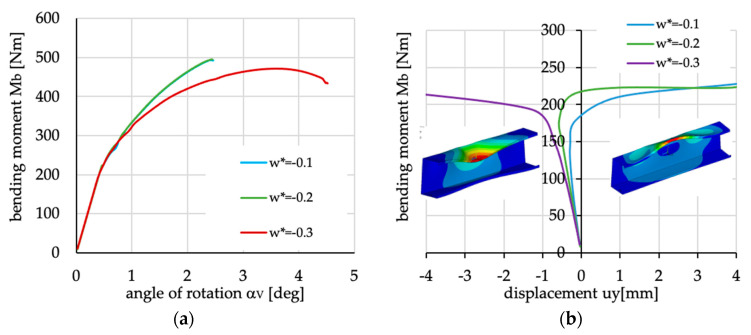
Bending moment vs. (**a**) angle of rotation and (**b**) upper flange displacement.

**Figure 31:**
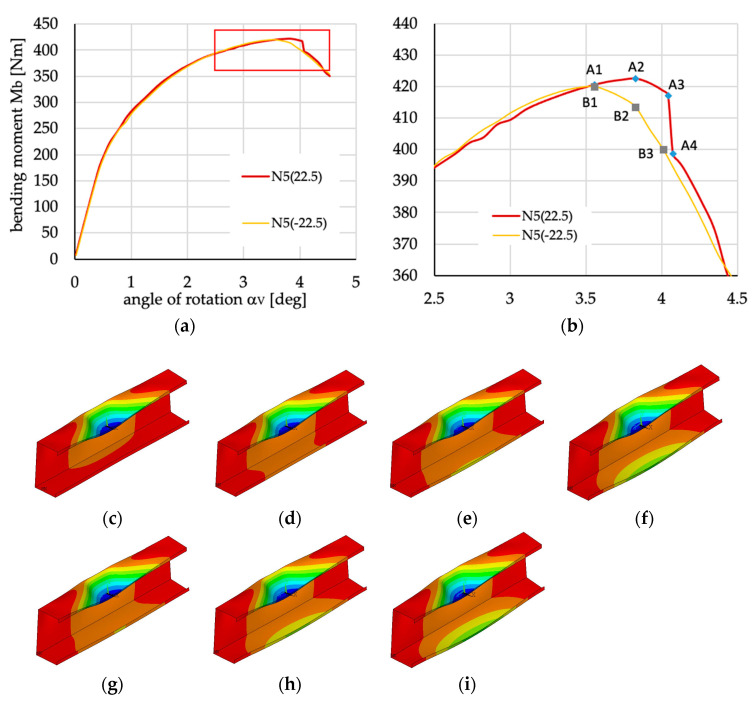
(**a**) Bending moment vs. angle of rotation α_V_. (**b**) Zoom of sub-figure (**a**). Shape of beam deflection from points depicted in sub-figure (**b**): (**c**) from point A1; (**d**) from point A2; (**e**) point A3, (**f**) point A4, (**g**) point B1, (**h**) point B2, (**i**) point B3.

Small differences in the course of curves presenting bending moment vs. angle of rotation α_V_ for both cases of beam cross-sections (cf. Figure 29) and layups N5(±22.5) need further analysis to explain why they appear. The analysis has been performed considering the lipped section beam, and the results are presented in Figure 31. As can be noted, the course of curves for beams N5(-22.5) and N5(22.5) (cf. Figure 31) depends on beam deflection, which influences its stiffness.

In the case of N5(-22.5), the flange under tension is bent with lower load (change from point B1 to B2) than in case N5(22.5)—points A1 and A2 in Figure 31. This can be possibly explained by the differences in the sign of the coupling stiffness matrix elements B_11_, B_22_, B_16_ and B_26_ and the fact that it is only in this case that the extension-bending, extension-twisting and shearing-bending load response coupling exist (**B***_l_*_t_).

## 7. Conclusions

The proposed numerical model has been validated by experimental tests of the channel section beam made of laminate with symmetrical layer arrangements. The parametric study was performed by employing the developed model. Channel and lipped section beams made of laminates with sixty different layer arrangements have been analyzed. Based on the obtained results, the following conclusion can be drawn:

- The coupling stiffness matrix element B_ij_, as well as elements D_16_ and D_26_, have no influence on the values of maximal buckling load and ultimate load for analyzed beams subjected to pure bending. It was found that only in the case of minimal buckling load and minimal ultimate load elements B_16_ and B_26_ have extremal values (cf. Table 6 and Table 7).
- The values of the buckling loads for lipped beams are over three-fold greater than for channel beams. This shows the significant influence of stiffeners on beam behavior. Ultimate load differs around 30%.
- The highest values of load-carrying capacity were obtained for 16-layer beams, which gives more flexibility for tailoring—more flexibility of coupling behavior and HTCS laminate design.
- In the case of the considered type of load, the ABD laminate stiffness matrix is not the only factor to influence the buckling and ultimate loads, but also the considered cross-section.

Summing up all of the above, it can be added that laminate tailoring depends not only on layer arrangement but also on the type of structure even if the load is the same. The different influence of layer arrangements on the behavior of channel and lipped section beams subjected to pure bending has been found.

## Figures and Tables

**Table 1:** The material properties laminate under consideration.

	E_1_	E_2_	G_12_	ν_12_	X^T^	Y^T^	X^C^	Y^C^	S
	[GPa]	[GPa]	[GPa]	[-]	[MPa]	[MPa]	[MPa]	[MPa]	[MPa]
**Data**	39.0	9.0	2.7	0.28	1250	43	620	140	112
**SD**	0.4	0.7	0.1	0.003	78	4	62	5	1

**Table 2:** Cross-section dimension of channel section beams for experimental tests.

Layer Arrangement	[0/90/0/90]_S_			[45/−45/90/0]_S_			[45/−45/45/−45]_S_		
Specimen No.	B_1_	B_2_	T	B_1_	B_2_	T	B_1_	B_2_	T
	[mm]	[mm]	[mm]	[mm]	[mm]	[mm]	[mm]	[mm]	[mm]
1	82.1	41.0	1.18	82.3	41.1	1.15	82.4	41.2	1.15
2	82.1	41.0	1.20	82.3	41.0	1.17	82.3	41.2	1.15
3	82.1	41.0	1.18	82.3	41.1	1.16	82.3	41.4	1.17
average:	82.1	41.00	1.19	82.3	41.1	1.16	82.3	41.3	1.16

**Table 3:** Notation of ABD matrix components of laminates under consideration.

Subscript Notation ESDU (1994) [40]	Description of Load Response Coupling	Stiffness Submatrices
**A_S_**	simple laminate no in-plane coupling	$\left[\begin{array}{ccc} A_{11} & A_{12} & 0\\ A_{12} & A_{22} & 0\\ 0 & A_{26} & A_{66} \end{array}\right]$
**A_F_**	shear-extension coupling	$\left[\begin{array}{ccc} A_{11} & A_{12} & A_{16}\\ A_{12} & A_{22} & A_{26}\\ A_{16} & A_{26} & A_{66} \end{array}\right]$
**B_t_**	extension-twisting and shear-bending	$\left[\begin{array}{ccc} 0 & 0 & B_{16}\\ 0 & 0 & B_{26}\\ B_{16} & B_{26} & 0 \end{array}\right]$
**B*_l_***	extension-bending	$\left[\begin{array}{ccc} B_{11} & 0 & 0\\ 0 & B_{22} & 0\\ 0 & 0 & 0 \end{array}\right]$
**B*_lt_***	extension-bending; extension-twisting; shearing-bending	$\left[\begin{array}{ccc} B_{11} & 0 & B_{16}\\ 0 & B_{22} & B_{26}\\ B_{16} & B_{26} & 0 \end{array}\right]$
**B_S_**	extension-bending and shear-twisting	$\left[\begin{array}{ccc} B_{11} & B_{12} & 0\\ B_{12} & B_{22} & 0\\ 0 & 0 & B_{66} \end{array}\right]$
**B_F_**	all in-plane with out-of-plane coupling	$\left[\begin{array}{ccc} B_{11} & B_{12} & B_{16}\\ B_{12} & B_{22} & B_{26}\\ B_{16} & B_{26} & B_{66} \end{array}\right]$
**D_S_**	simple laminate no out-of-plane coupling	$\left[\begin{array}{ccc} D_{11} & D_{12} & 0\\ D_{12} & D_{22} & 0\\ 0 & 0 & D_{66} \end{array}\right]$
**D_F_**	twisting-bending coupling	$\left[\begin{array}{ccc} D_{11} & D_{12} & D_{16}\\ D_{12} & D_{22} & D_{26}\\ D_{16} & D_{26} & D_{66} \end{array}\right]$

**Table 4:** The layer arrangements of laminates under consideration.

Case ID	Number of Layers	Layer Arrangement	Laminate Type [40]	Considered θ [deg]
S1	8	45/−45/45/−45/−45/45/−45/45	A_S_B_0_D_F_	-
S2	8	0/90/0/90/90/0/90/0	A_S_ B_0_D_S_	-
S3	8	45/−45/90/0/0/90/−45/45	A_S_ B_0_D_F_	-
N1	8	45/45/45/45/−45/−45/−45/−45	A_S_B_t_D_S_	-
N2	8	90/90/90/90/0/0/0/0	A_S_B_l_D_S_	-
A1(θ) [41]	8	θ/(θ−90)_2_/θ/−θ/(90−θ)_2_/−θ	A_S_B_t_D_S_	±22.5
A2(θ)	8	0/90/θ/90−θ/θ−90/−θ/0/90	A_S_B*_lt_*D_S_	±35, ±45, ±55
A2R(θ)	8	90/0/−θ/θ−90/90−θ/θ/90/0	A_S_B*_lt_*D_S_	−35, −45, −55
N3(θ) [1]	8	90/0/θ/−θ/0/90/−θ/θ	A_S_B_S_D_F_	±30, ±45, ±60
N3R(θ)	8	θ/−θ/90/0/−θ/θ/0/90	A_S_B_S_D_F_	−30, −45, −60
N4(θ)	8	45/−45/45/−45/−θ/90−θ/θ/θ−90	A_S_B_F_D_F_	0, 90, ±22.5
N4R(θ)	8	θ−90/θ/90−θ/−θ/−45/45/−45/45	A_S_B_F_D_F_	90, 22.5
N5(θ)	8	45/−45/θ/−θ/θ−90/90−θ/−45/45	A_S_B*_lt_*D_F_	±22.5
N6(θ)	8	45/−45/θ−90/θ/−θ/90−θ/−45/45	A_S_B*_t_*D_F_	±26.5, ±45, ±63.5
N16(θ) [1]	16	−θ/90/θ/0/0/θ/0/0/90/−θ/0/−θ/θ/0/−θ/θ	A_F_B_F_D_F_	±30, ±45, ±60
N16R(θ)	16	θ/−θ/0/θ/−θ/0/−θ/90/0/0/θ/0/0/θ/90/−θ	A_F_B_F_D_F_	−30, −45, −60

**Table 5:** Buckling and ultimate loads with corresponding angles of rotation for considered cases.

Case ID	θ	C-Section Beam				Lipped Section Beam			
		M_cr_	M_u_	(α_V_)_u_	(α_H_)_u_	M_cr_	M_u_	(α_V_)_u_	(α_H_)_u_
		[Nm]	[Nm]	[deg]	[deg]	[Nm]	[Nm]	[deg]	[deg]
**S1**	–	78.4	288.5	**2.94**	**3.63**	190.6	352.8	4.01	4.15
**S2**		60.8	367.2	1.96	2.88	**291.4**	502.6	3.22	2.96
**S3**		76.4	358.9	2.12	2.65	248.5	477.9	3.46	3.39
A1(θ)	±22.5	60.8	335.7	2.15	3.07	223.8	449.2	3.37	3.27
A2(θ)	±35	59.7	354.6	2.16	2.89	236.3	487.7	3.94	3.6
	±45	59.9	350.8	2.23	2.99	233.1	475.6	3.83	3.58
	±55	59.4	354.6	2.16	2.89	238.8	485.6	3.74	3.45
A2R(θ)	±35	59.8	354	2.16	2.96	241.2	482.7	3.55	3.34
	±45	59.9	350.1	2.23	3.07	238.3	475.3	3.64	3.46
	±55	59.5	354.3	2.16	2.96	243.8	485.2	3.65	3.47
N1	–	58.2	**250.7**	2.67	3.23	**160.3**	**311.5**	3.92	3.89
N1R		58.3	**250.8**	2.67	3.19	**160.3**	**311.6**	3.92	3.86
N2		52.4	329.3	1.87	3.09	239.4	477.9	3.15	2.96
N2R		**45.8**	319.2	**1.81**	2.56	216	460.5	3.71	3.05
N3(θ)	±30	66	369.5	1.96	2.96	267.2	512.2	3.15	3.09
	±45	66.2	338.8	2.12	2.92	246.8	462.7	3.46	3.43
	±60	61.6	320.5	2.26	2.96	240.4	444.1	3.98	3.79
N3R(θ)	±30	65.7	396	2.16	3.15	263.6	512.3	3.15	3.08
	±45	66	367	2.32	3	248.6	499.4	4.1	3.89
	±60	61.5	341.4	2.42	3.11	246.9	472	**4.5**	**4.22**
N4(θ)	0	58.4	357.9	2.28	3.08	234.1	–	–	–
	90	60	361.7	2.39	3.35	225.9	–	–	–
	22.5	71.1	340.3	2.53	3.14	220.4	436	4.01	3.96
	−22.5	69.9	336.7	2.61	3.33	219.2	434.4	4.04	3.99
N4R(θ)	90	60.2	307.3	2.05	3.02	218.3	410	3.14	3.38
	22.5	70.1	308.8	2.32	3.11	221	405.3	3.46	3.65
N5(θ)	±22.5	76.7	329.9	2.53	3.21	219.4	421.2	3.69	3.74
N6(θ)	±26.5	77.2	322.1	2.53	3.18	215.6	410.2	3.74	3.79
	±45	**79.1**	289.6	2.94	3.6	191	353.8	4.01	4.15
	±63.5	77.6	321.1	2.53	3.15	210.6	403.7	3.74	3.82
N16(θ)	±30	71.4	385	1.82	2.76	270.2	**548.6**	**2.87**	**2.76**
	±45	73.8	370.6	1.98	2.74	262.8	529.5	3.08	2.9
	±60	68.4	361.7	2.02	2.6	260.4	518	3.15	2.95
N16R(θ)	±30	71.2	**405.5**	2	2.93	265.8	543.6	3.09	2.81
	±45	73.7	391.4	2.16	2.92	258.9	522.2	3.46	3.24
	±60	68.3	371.4	2.03	**2.42**	262.8	515.9	3.74	3.52

**Table 6:** Nondimensional value of ABD laminate stiffness matrix for laminates used in channel section beam for which the extremal buckling and ultimate load were obtained.

Sub-Matrix	Minimal M_cr_	Maximal M_cr_	Minimal M_u_	Maximal M_u_
	N2R	N6(±45)	N1, N1R	N16R(±30)
**A**	$\left[\begin{array}{ccc} 0.84 & 0.24 & 0\\ & 0.97 & 0\\ & & 0.25 \end{array}\right]$	$\left[\begin{array}{ccc} 0.56 & 1 & 0\\ & 0.64 & 0\\ & & 1 \end{array}\right]$	$\left[\begin{array}{ccc} 0.56 & 1 & 0\\ & 0.64 & 0\\ & & 1 \end{array}\right]$	$\left[\begin{array}{ccc} 1 & 0.52 & -1\\ & 0.54 & -0.30\\ & & 0.53 \end{array}\right]$
**B**	$\left[\begin{array}{ccc} -1 & 0 & 0\\ & 1 & 0\\ & & 0 \end{array}\right]$	$\left[\begin{array}{ccc} 0 & 0 & 0.12\\ & 0 & 0.12\\ & & 0 \end{array}\right]$	$\left[\begin{array}{ccc} 0 & 0 & -1\\ & 0 & -1\\ & & 0 \end{array}\right]$	$\left[\begin{array}{ccc} -0.07 & -0.19 & 0.06\\ & 0.18 & 0.02\\ & & -0.19 \end{array}\right]$
**D**	$\left[\begin{array}{ccc} 0.81 & 0.24 & 0\\ & 0.87 & 0\\ & & 0.25 \end{array}\right]$	$\left[\begin{array}{ccc} 0.54 & 1 & 0.75\\ & 0.58 & 0.62\\ & & 1 \end{array}\right]$	$\left[\begin{array}{ccc} 0.54 & 1 & 0\\ & 0.58 & 0\\ & & 1 \end{array}\right]$	$\left[\begin{array}{ccc} 0.89 & 0.62 & -0.05\\ & 0.50 & -0.01\\ & & 0.62 \end{array}\right]$

**Table 7:** Nondimensional value of ABD laminate stiffness matrix for laminates used in lipped section beam for which the extremal buckling and ultimate load were obtained.

Sub-Matrix	Minimal M_cr_	Maximal M_cr_	Minimal M_u_	Maximal M_u_
	N1, N1R	S2	N1	N16(±30)
**A**	$\left[\begin{array}{ccc} 0.56 & 1 & 0\\ & 0.64 & 0\\ & & 1 \end{array}\right]$	$\left[\begin{array}{ccc} 0.84 & 0.24 & 0\\ & 0.97 & 0\\ & & 0.25 \end{array}\right]$	$\left[\begin{array}{ccc} 0.56 & 1 & 0\\ & 0.64 & 0\\ & & 1 \end{array}\right]$	$\left[\begin{array}{ccc} 1 & 0.52 & 1\\ & 0.54 & 0.30\\ & & 0.53 \end{array}\right]$
**B**	$\left[\begin{array}{ccc} 0 & 0 & -1\\ & 0 & -1\\ & & 0 \end{array}\right]$	$\left[\begin{array}{ccc} 0 & 0 & 0\\ & 0 & 0\\ & & 0 \end{array}\right]$	$\left[\begin{array}{ccc} 0 & 0 & -1\\ & 0 & -1\\ & & 0 \end{array}\right]$	$\left[\begin{array}{ccc} 0.07 & 0.19 & 0.06\\ & -0.18 & 0.02\\ & & 0.19 \end{array}\right]$
**D**	$\left[\begin{array}{ccc} 0.54 & 1 & 0\\ & 0.58 & 0\\ & & 1 \end{array}\right]$	$\left[\begin{array}{ccc} 1 & 0.24 & 0\\ & 0.67 & 0\\ & & 0.25 \end{array}\right]$	$\left[\begin{array}{ccc} 0.54 & 1 & 0\\ & 0.58 & 0\\ & & 1 \end{array}\right]$	$\left[\begin{array}{ccc} 0.89 & 0.62 & 0.05\\ & 0.50 & 0.01\\ & & 0.62 \end{array}\right]$
